# Plumbagin Enhances the Anticancer Efficacy of Cisplatin by Increasing Intracellular ROS in Human Tongue Squamous Cell Carcinoma

**DOI:** 10.1155/2020/5649174

**Published:** 2020-03-25

**Authors:** Danfeng Xue, Shu-Ting Pan, Xiongming Zhou, Fangfei Ye, Qun Zhou, Fanzhe Shi, Fei He, Hui Yu, Jiaxuan Qiu

**Affiliations:** ^1^Department of Oral and Maxillofacial Surgery, The First Affiliated Hospital of Nanchang University, Nanchang, 330006 Jiangxi, China; ^2^Department of Pathology, The First Affiliated Hospital of Nanchang University, Nanchang, 330006 Jiangxi, China

## Abstract

Cisplatin is widely used in the treatment of tongue squamous cell carcinoma (TSCC), but its clinical efficacy is limited by drug resistance and toxic side effects. Hence, a novel compound capable of enhancing the anticancer effect of cisplatin while reducing the side effects is urgently needed. We have previously shown that plumbagin (PLB), an anticancer phytochemical, is able to inhibit the growth of TSCC *in vitro* and *in vivo*. The objective of this study was to investigate the effect of PLB in reversing the resistance of TSCC to cisplatin as well as its molecular mechanisms. Here, we found that PLB enhances cisplatin-induced cytotoxicity, apoptosis, and autophagy in CAL27 and cisplatin-resistant CAL27/CDDP cells. PLB could inhibit the viability and growth of TSCC cells by increasing the production of intracellular reactive oxygen species (ROS). In addition, the combination treatment of PLB and cisplatin resulted in a synergistic inhibition of TSCC viability, apoptosis, and autophagy by increasing intracellular ROS, which may be achieved by activating JNK and inhibiting AKT/mTOR signaling pathways. Finally, the synergistic treatment was also demonstrated *in vivo*. Therefore, PLB combined with cisplatin is a potential therapeutic strategy against therapy TSCC cisplatin resistance.

## 1. Introduction

Tongue squamous cell carcinoma (TSCC), the most common malignant tumor of the oral region, is well known for its high rate of lymph node metastasis and poor prognosis [1, 2]. Currently, combination chemotherapy based on cisplatin (cis-diamminedichloroplatinum II, CDDP) is the standard treatment for many types of cancers, including TSCC. However, its effectiveness is often limited as a result of drug resistance and severe side effects [3]. In the past few decades, despite the significant development in understanding the chemotherapy resistance mechanisms, no substantial progress has been made in overcoming cisplatin resistance. Therefore, it is urgent to explore a novel therapeutic strategy to reverse the resistance of cisplatin while minimizing the toxic effect on patients.

Phytochemicals are naturally occurring plant-derived compounds that have been widely used to treat a variety of malignant tumors due to their availability, biological activity, and nontoxic effects [4]. Emerging evidence suggests that numerous phytochemicals can reverse the resistance of various malignancies to cisplatin while counteracting organ toxicity caused by cisplatin [5–7]. Thus, in many types of malignancies, phytochemicals are candidates for overcoming cisplatin resistance. Plumbagin (5-hydroxy-2-methyl-1,4-naphthoquinone, PLB) is a yellow crystalline phytochemical isolated from the roots of plant *Plumbago zeylanica L*. [8]. It exhibits anticancer effects in human cancers by modulating several molecular mechanisms including autophagy, apoptosis, cell cycle arrest, and generation of reactive oxygen species (ROS) [9, 10]. Our previous studies found that PLB has a significant inhibitory effect on the proliferation of TSCC *in vitro* and *in vivo* and induces the production of ROS in TSCC SCC25 cell line [ 11, 12]. However, whether PLB can enhance the sensitivity of TSCC to cisplatin remains to be explored.

ROS is an active form of oxygen, and its induced cytotoxicity is an important mechanism for cisplatin to kill tumor cells [13]. Cisplatin induces high ROS levels that cause cancer cell apoptosis [14]. In addition, ROS can indirectly regulate CDDP-induced apoptosis and autophagy and exhibit potential chemosensitization in various malignancies including cholangiocarcinoma [15], lung cancer [16], and malignant mesothelioma [17]. Mechanism studies have revealed that plumbagin induces cytotoxicity in various cancers, such as cervical cancer [18] and breast cancer [19], by producing ROS. Studies have also shown that plumbagin can be used in combination with existing anticancer drugs, which will help treat patients that are chemotherapy-resistant [20].

Therefore, we hypothesized that ROS is closely related to cisplatin resistance in TSCC. In addition, the combination of PLB and CDDP could exhibit a synergistic anticancer effect by increasing the production of ROS. In the present study, we investigated for the first time the role of PLB in reversing cisplatin resistance in TSCC and its underlying mechanism. This study will provide a new treatment option for cisplatin resistance in TSCC.

## 2. Materials and Methods

### 2.1. Reagents and Chemicals

Plumbagin (5-hydroxy-2-methyl-1,4-naphthoquinone) was purchased from Sigma-Aldrich (St. Louis, MO, USA). Cisplatin was purchased from Qilu Pharm (Jinan, China). 3-Methyladenine (3-MA) and N-acetylcysteine (NAC) were obtained from MedChem Express (Shanghai, China). Fetal bovine serum (FBS) and Dulbecco's modified Eagle's medium (DMEM) high glucose were purchased from Gibco (Carlsbad, CA, USA). The antibodies used included Bax (5023), Bcl-2 (4223), cleaved caspase-3 (9664), Beclin-1 (3495), LC3B (3868), SQSTM1/p62 (39749), SAPK/JNK (9252), phospho-SAPK/JNK (Thr 183/Try 185, 4668), phospho-AKT (Ser 473, 4060), and phospho-mTOR (Ser 2448, 5536), all of which were acquired from Cell Signaling Technology (Danvers, MA, USA). Mouse anti-human GAPDH, rabbit anti-human *β*-actin, and secondary antibodies including goat anti-rabbit and goat anti-mouse were purchased from Proteintech (Wuhan, China).

### 2.2. Cell Culture

Human tongue squamous cell carcinoma cell line (CAL27) was obtained from the Cell Bank of the Chinese Academy of Sciences (Shanghai, China). Cisplatin-resistant cell line (CAL27/CDDP) was provided by Professor Jinsong Li, Sun Yat-sen Memorial Hospital (Guangzhou, China). The stable cisplatin-resistant line CAL27/CDDP was established by exposing CAL27 to cisplatin at the indicated time [21]. Both types of TSCC cells were maintained in DMEM high glucose medium supplemented with 10% FBS, 100 units/mL penicillin, and 100 *μ*g/mL streptomycin. Cells were maintained under a humidified atmosphere at 37°C with 5% CO_2_.

### 2.3. Cell Viability Assays

The cell viability of TSCC cells was measured using Cell Counting Kit-8 (CCK-8, Zomanbio, Beijing, China). CAL27 and CAL27/CDDP cells were seeded in 96-well plates at a density of 1 × 10^4^ cells per well. Cells were exposed to different concentrations of CDDP and PLB alone and their combination for 24 h to determine the individual and combined effects of CDDP and PLB. To determine the role of ROS in PLB-induced cytotoxicity effects, TSCC cells were incubated with various concentrations of PLB (2.5, 5, 10, 20, and 40 *μ*M) for 24 h alone or pretreated with ROS scavenger NAC (5 mM) for 1 h. To evaluate the role of ROS in PLB combined CDDP-induced cytotoxicity effects, TSCC cells were treated with NAC (5 mM), PLB (5 *μ*M) combined with CDDP (16.7 *μ*M), and preincubated with NAC for 1 h before exposure to PLB combined with CDDP. To assess the role of autophagy in PLB- or cisplatin-induced cytotoxicity effects, cells were exposed to autophagy inhibitor 3-MA (2 mM), PLB (5 *μ*M) and CDDP (16.7 *μ*M) alone, and pretreatment with 3-MA for 1 h prior to PLB or CDDP. After different treatments, 10 *μ*L of CCK-8 was added to each well and incubated for 1 h at 37°C. The optical density (OD) of each well was measured at a wavelength of 450 nm using a microplate reader (Tecan, Switzerland). Cell viability was calculated using the following formula: Cell viability (%) = (OD treated − OD blank)/(OD control − OD blank) × 100%. The half maximal inhibitory concentration (IC_50_) value and the data were calculated by Prism 6 software (GraphPad, San Diego, CA, USA) and expressed as the percentage cell viability relative to the control.

### 2.4. Combination Index

The combination effect of PLB and CDDP on TSCC cells was evaluated using the combination index (CI) according to the median dose-effect analysis by Chou and Talalay [22]. CI analysis was performed using CompuSyn Software (ComboSyn, Inc., Paramus, NJ, USA). The combined effect is represented as follows: CI < 1 represents a synergistic effect; CI = 1 indicates an additive cytotoxicity; and CI > 1 represents an antagonistic effect.

### 2.5. DAPI Staining

Nuclear condensation and fragmentation were observed by staining nuclei with DAPI (Beyotime, Shanghai, China). Cells were seeded at a density of 1 × 10^5^ cells/well in 6-well plates and incubated with CDDP (16.7 *μ*M) and PLB (5 *μ*M) alone and their combination for 24 h. The attached cells were washed with PBS for three times and fixed with 4% paraformaldehyde at room temperature for 15 min. Then, cells were stained with DAPI solution (1 mg/mL) for 15 min at 37°C in the dark. After treatment, cells were washed twice with PBS and then observed using fluorescence microscope (×400 magnification, Olympus, Japan). Apoptotic nuclei can be identified by the fragments of nucleosomes or concentrated chromatin and marked with yellow arrows.

### 2.6. Flow Cytometric Analysis of Apoptosis

Cells in the early and late stages of apoptosis were measured by flow cytometry using the Annexin V-FITC apoptosis detection kit (BD Biosciences, USA). Briefly, cells were cultured in 6 cm dishes at a density of 3 × 10^5^ cells per dish and incubated overnight for adherence. Then, the cells were exposed to CDDP (16.7 *μ*M) and PLB (5 *μ*M) alone and their combination for 24 h. To determine the role of ROS in the combination of PLB and CDDP treatment-induced cytotoxicity, cells were exposed to NAC (5 mM), PLB (5 *μ*M) combined with CDDP (16.7 *μ*M), and preincubation with NAC for 1 h before exposure to PLB combined with CDDP. To assess the role of autophagy in PLB- or CDDP-induced apoptosis effects, cells were exposed to autophagy inhibitor 3-MA (2 mM), PLB (5 *μ*M) and CDDP (16.7 *μ*M) alone, and pretreatment with 3-MA for 1 h prior to PLB or CDDP. After treatment, the cells were trypsinized and collected by 1000 rpm/min for 5 min. The centrifuged cells were washed twice with ice-cold PBS and resuspended in the 1x binding buffer containing Annexin V-FITC and PI for 15 min at 37°C in the dark. Finally, samples were detected within 30 min by using a Flow Cytometer (Beckman Coulter, Brea, CA, USA). Both early and late apoptotic cells were recorded as apoptotic cells, and the results are expressed as the percentage of total cells.

### 2.7. LysoTracker Red Staining

Cells were seeded at a density of 2.5 × 10^4^ cells/well on 14 mm coverslips in 24-well plates and treated with CDDP (16.7 *μ*M), PLB (5 *μ*M), and their combination for 24 h. Then, cells were washed with PBS for three times and incubated with 50 nM of LysoTracker Red DND-99 (Invitrogen™, Oregon, USA) at 37°C for 30 min in the dark. After that, coverslips were removed and mounted on a glass slide. Images were taken by a confocal laser scanning microscope (Zeiss, LSM 880, Germany).

### 2.8. Transmission Electron Microscopy

CAL27 cells were seeded into a 6-well plate at a density of 1 × 10^5^ cells/well and treated with CDDP (16.7 *μ*M), PLB (5 *μ*M), and their combination for 24 h. After treatment, cells were fixed in 2.5% glutaraldehyde for 2 h at room temperature and washed 3 times each for 20 min with PBS. The cells were then postfixed in 1% aqueous osmium tetroxide at 4°C for 2 h and washed 3 times each for 20 min with PBS. Then, the samples were dehydrated with series of grade alcohol (50%-100%) and infiltrated twice with propylene oxide for 12 h each. Samples were embedded in epoxy resin and polymerized at 60°C for 2 days. Ultrathin sections were cut at 600 nm thickness. Images were taken using a TECNAI 20 TWIN transmission electron microscope.

### 2.9. Western Blot Analysis

After treatment with different regimens, cell samples were lysed with RIPA buffer (Beyotime, Shanghai, China) containing protease and phosphatase inhibitor cocktails for 30 min at 4°C. Then, the lysate was centrifuged at 12,000 rpm/min for 15 min at 4°C, and the supernatant was collected. Total protein was determined using the BCA protein assay kit (Thermo Fisher Scientific, Waltham, MA, USA). Protein samples were separated on 8%–15% sodium dodecyl sulfate polyacrylamide gel electrophoresis (SDS-PAGE) and transferred to PVDF membranes (Millipore, Bedford, MA, USA). After incubation in 5% skim milk or 5% BSA at room temperature for 2 h, the membrane was incubated with the corresponding primary antibody at 4°C overnight. Then, membranes were washed by Tris-buffered saline with Tween-20 (TBST) and incubated with the respective secondary antibody for 1 h at room temperature. The membrane was washed using TBST and captured using the Bio-Rad system (Bio-Rad Laboratories Inc., Hercules, CA, USA).

### 2.10. Clone Formation Assay

TSCC cells were seeded in six-well plates at a density of 500 cells/well evenly. After 24 h, cells were incubated with various concentrations of PLB (0, 1.25, 2.5, and 5 *μ*M) alone or pretreated with ROS scavenger NAC (5 mM) for about 14 days until most of the colony contained more than 50 cells. Then, the medium was discarded. Cells were washed with PBS, fixed with 4% paraformaldehyde, and stained with 0.1% crystal violet for 30 min at room temperature. The counts of cell colonies were calculated manually.

### 2.11. Measurement of Intracellular ROS Generation and Mitochondrial Superoxide

Intracellular ROS generation was detected by fluorescence microscopy using 2′,7′-dichlorofluorescein diacetate (DCFH-DA) fluorescent probe (Beyotime, Shanghai, China). Cells were seeded in 6-well plates at a density of 1 × 10^5^ cells/well overnight to allow adherence. To evaluate ROS generation in a dose-dependent manner, CAL27 and CAL27/CDDP cells were treated with different concentrations of PLB (0, 2.5, 5, and 10 *μ*M) for 24 h alone or pretreated with ROS scavenger NAC (5 mM) for 1 h. To evaluate the role of ROS in the combination of PLB and CDDP treatment-induced cytotoxicity, CAL27 and CAL27/CDDP cells were treated with NAC (5 mM), PLB (5 *μ*M) combined with CDDP (16.7 *μ*M), and NAC for 1 h prior to PLB combined with CDDP. Then, the cells were washed with PBS for three times and incubated in serum-free medium containing 10 *μ*M DCFH-DA for 30 min at 37°C in the dark. Next, the excess DCFH-DA was removed, and the cells were washed with PBS three times. Intracellular ROS production was quantified by measuring the intracellular accumulation of dichlorofluorescein (DCF) which is caused by the oxidation of DCFH_2_. The DCF-stained cells were observed using a fluorescence microscope (×200 magnification, Olympus, Japan).

Mitochondrial superoxide level in cells was investigated with the MitoSOX™ Red Mitochondrial Superoxide Dye Kit (Invitrogen™ Oregon, USA) by a fluorescence microscope. Briefly, cells were seeded at a density of 1 × 10^5^ cells/well in 6-well plates overnight to allow adherence. The treatment method is the same as described above. Next, the cells were stained with 4 *μ*M MitoSOX Red dye at 37°C for 30 min in the dark. The level of ROS was measured by a fluorescence microscope (×200 magnification, Olympus, Japan). Fluorescence intensity of individual cells was analyzed by the software ImageJ.

### 2.12. Xenograft Tumor Growth Studies

All male BALB/c nude mice (certificate no. SCXK 2016-0002) with body weights approximately 20 g were purchased from SLAC Jingda Experimental Animal Co., Ltd (Hunan, China). All protocols were approved by the Ethics Committee of Nanchang University (permit no. SYXK2015-0003). All efforts were made to minimize animal suffering. Mice were kept on air-conditioned rooms under controlled light (12 h light : 12 h dark) and received sterilized food and water in a temperature- and humidity-controlled environment. Each mouse was injected with 0.15 mL of cell suspension containing 1 × 10^6^ CAL27/CDDP cells into the right flanks. After tumor volumes reached approximately 80 mm^3^, 24 mice were divided randomly into four groups (*n* = 6 in each group). (1) Control group: mice were injected with 0.9% saline. (2) PLB group: mice were injected with 3 mg/kg PLB every other day. (3) CDDP group: mice were injected with 4 mg/kg CDDP every three days. (4) PLB+CDDP group, combinational group: both PLB and CDDP were administered according to the aforementioned regimens. Body weight and tumor size were measured every day. Tumor volumes were calculated according to the following formula: *ab*^2^ × 1/2 (*a* is the largest diameter, and *b* is the smallest diameter of the tumor). At the end of 21 days, all mice were sacrificed by cervical dislocation, and the primary tumors were removed and weighted. Major organs including the heart, liver, spleen, lung, and kidney were removed and fixed in 10% formalin for histopathological studies. After fixation, tissues were dehydrated in a series of gradients of ethanol and xylene, embedded in paraffin, cut into thin slices, and then stained with hematoxylin and eosin (H&E). H&E-stained sections were examined under a light microscope at a magnification of ×400.

### 2.13. Immunohistochemistry

After treatment *in vivo*, immunohistochemistry (IHC) was performed to evaluate Ki-67 expression in xenograft tumor tissues. All tumor tissues were separated and fixed with 4% paraformaldehyde. After 48 h, tissues were embedded in paraffin and cut into 4 *μ*m sections. Slides were blocked and incubated with antibodies targeting Ki-67 (1 : 200) at 4°C overnight. Then, put the slide with secondary antibody at room temperature for 30 min. Images were captured using a microscope, and Ki-67 expression was evaluated by counting the number of positive cells from 5 randomly selected fields under a light microscope at a magnification of ×400. Data are presented as the percentage of positive cells.

### 2.14. Statistical Analysis

Data are reported as the mean ± standard deviation (SD). One-way analysis of variance (ANOVA) was used to compare differences of multiple groups of values. *p* < 0.05 was considered significant. All statistical analyses were performed using Prism 6.0 (GraphPad, San Diego, CA, USA).

## 3. Results

### 3.1. Plumbagin Synergistically Enhances the Cytotoxicity of Cisplatin in TSCC Cells

**Figure 1:**
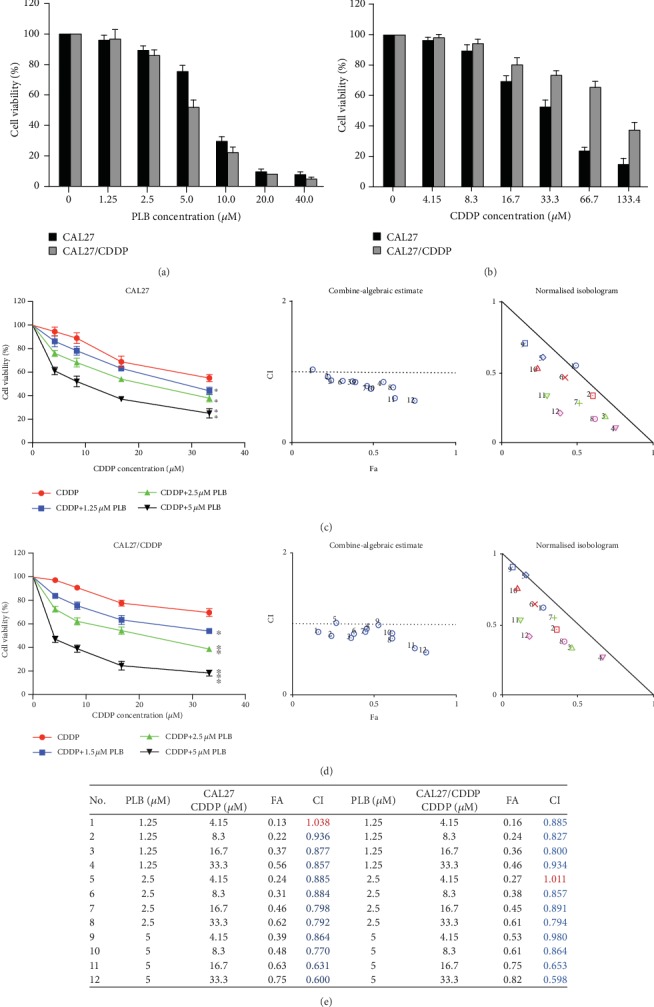
Effect of PLB and CDDP alone and their combination on the viability of TSCC cells. (a, b) CAL27 and CAL27/CDDP cells were treated with different concentrations of PLB or CDDP for 24 h, and the cell viability was determined by CCK-8 assay. (c, d) CAL27 and CAL27/CDDP cells were treated with different combinations of concentrations of PLB and CDDP for 24 h, and the cell viability was determined by CCK-8 assay. The CI values of PLB combined with CDDP were calculated for the two TSCC cell lines. (e) The data are presented as the mean ± SD. ^∗^*p* < 0.05, ^∗∗^*p* < 0.01, and ^∗∗∗^*p* < 0.001 vs. CDDP.

The CCK-8 assay was used to examine the effects of PLB and CDDP alone and their combination on the viability of CAL27 and cisplatin-resistant CAL27/CDDP cells. As shown in Figures 1(a) and 1(b), both PLB treatment alone and CDDP treatment alone reduced the viability of the two TSCC cell lines in a dose-dependent manner. After 24 h treatment, the IC_50_ values of PLB were 7.374 *μ*M in CAL27 and 5.433 *μ*M in CAL27/CDDP cells, respectively. The IC_50_ values of CDDP for CAL27 and CAL27/CDDP were 33.08 *μ*M and 94.28 *μ*M, respectively. The result showed that CAL27 cells were much more sensitive to CDDP than CAL27/CDDP cells, as reflected by the low IC_50_ values. We then explored whether PLB could synergistically enhance CDDP-mediated cytotoxic effects in TSCC cells. We tested the effect of low concentrations of PLB (1.25, 2.5, and 5 *μ*M) combined with CDDP (4.15, 8.3, 16.7, and 33.3 *μ*M) on the viability of CAL27 and CAL27/CDDP cells. As illustrated in Figures 1(c) and 1(d), compared with CDDP treatment alone, the combination treatment significantly increased cytotoxicity in both CAL27 and CAL27/CDDP cells. In addition, CI values showed that the combination of PLB and CDDP exerted synergistic cytotoxic effects at almost all tested concentrations (Figure 1(e)). Our findings suggest that PLB combined with CDDP synergistically inhibits the viability of TSCC cells and has a better inhibition on CAL27/CDDP cells. Next, we selected 5 *μ*M PLB and 16.7 *μ*M CDDP for our subsequent experiments.

### 3.2. Plumbagin Enhanced the Proapoptosis Effect of Cisplatin in TSCC Cells via Caspase/Bax/Bcl-2 Signaling Pathway

**Figure 2:**
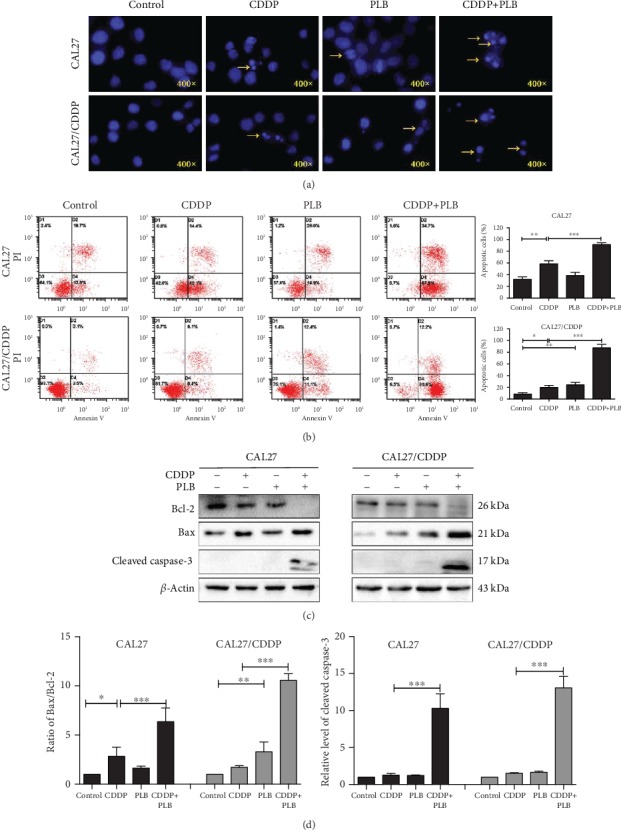
Apoptosis-inducing effects of PLB and CDDP in TSCC cells. CAL27 and CAL27/CDDP cells were treated with PLB (5 *μ*M) and CDDP (16.7 *μ*M) alone and their combination for 24 h. (a) Apoptosis nuclear morphological changes were evaluated by DAPI staining and observed under fluorescence microscopy. Yellow arrows indicate nuclear fragmentation. (b) Flow cytometry analysis of Annexin V and PI staining of apoptotic cells. The histograms indicate the quantification of the early and late apoptotic cells. (c, d) The expression levels of apoptosis-related proteins in two TSCC cells were measured by Western blotting. The histograms indicate the relative expression levels of proteins. The quantitative data are shown as the mean ± SD of 3 independent experiments. ^∗^*p* < 0.05, ^∗∗^*p* < 0.01, and ^∗∗∗^*p* < 0.001.

Our current research shows that PLB in combination with CDDP exhibits a synergistic effect in TSCC cells. Therefore, it is important to further explore the synergistic mechanism of PLB and CDDP cotreatment. In our previous study, we have demonstrated that PLB induces apoptosis in TSCC cells [12]. To further investigate the effect of PLB on CDDP-mediated apoptosis, the level of apoptosis in TSCC cells was detected after treatment with CDDP and PLB alone or their combination. Firstly, the nuclear morphological changes of both TSCC cells were detected by DAPI staining. As shown in Figure 2(a), the combination treatment dramatically caused nuclear fragmentation in both CAL27 and CAL27/CDDP cells compared to PLB or CDDP treatment alone. Next, we used Annexin V/PI double staining to quantify apoptosis. As shown in Figure 2(b), the combination treatment significantly increased both early and late apoptotic cells in CAL27 and CAL27/CDDP cells (91.33% and 87.4%, respectively), compared with CDDP (58.5% and 19.9%, respectively) or PLB (38.46% and 24.2%, respectively) treatment alone. The effect in which plumbagin enhanced the apoptosis induction of cisplatin in CAL27/CDDP cells was more potent than that in CAL27 cells. To explore the potential molecular mechanisms of the proapoptotic effects caused by CDDP and PLB, we tested the expression of apoptosis-related proteins including Bax, cleaved caspase-3, and Bcl-2 in two TSCC cells by Western blotting. As shown in Figures 2(c) and 2(d), the combination treatment significantly upregulated the level of proteins including proapoptotic proteins Bax and cleaved caspase-3, accompanied by obvious downregulation of the antiapoptotic protein Bcl-2 in both TSCC cells, compared with CDDP or PLB treatment alone. Taken together, these results indicated that PLB enhances the apoptosis-inducing effect of cisplatin in TSCC cells via the caspase/Bax/Bcl-2 signaling pathway.

### 3.3. Plumbagin Enhanced the Proautophagy Effect of Cisplatin in TSCC Cells through the AKT/mTOR Signaling Pathway

**Figure 3:**
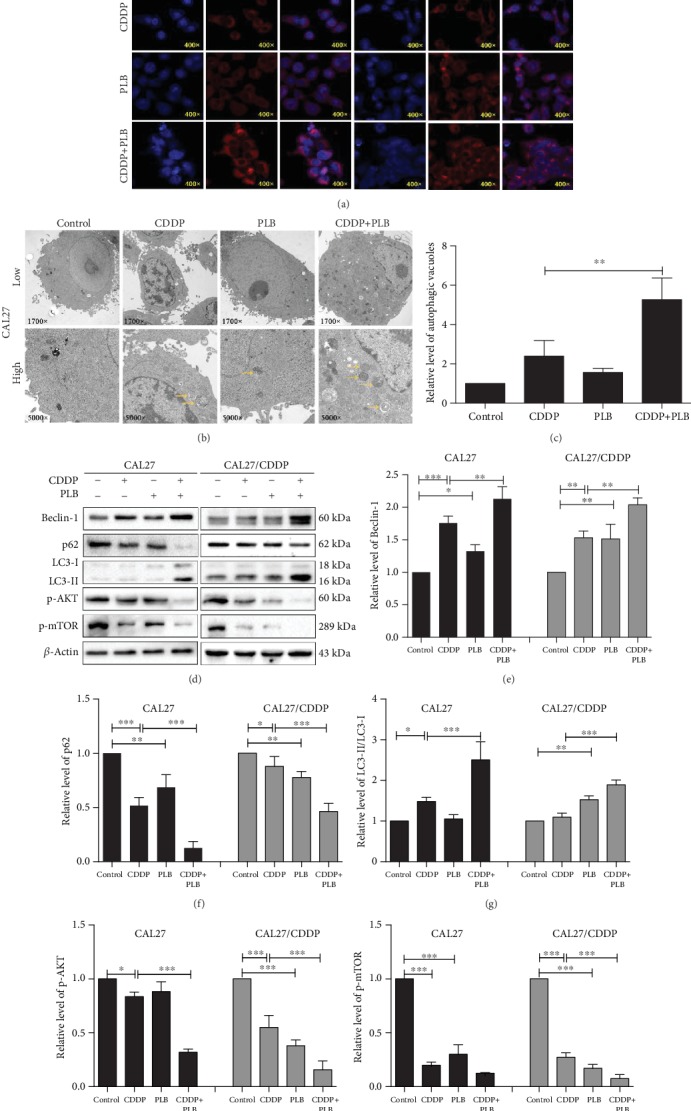
Autophagy-inducing effects of PLB and CDDP in TSCC cells. CAL27 and CAL27/CDDP cells were treated with PLB (5 *μ*M) and CDDP (16.7 *μ*M) alone and their combination for 24 h. (a) CAL27 and CAL27/CDDP cells incubated with 50 nM of LysoTracker Red DND-99. Red color intensity shows the acidic compartment of the cell, indicating lysosomes and autolysosomes, observed under a confocal laser scanning microscope. (b, c) Transmission electron microcopy (TEM) was used to evaluate the changes in autophagosomes in CAL27 cells. Yellow arrows indicate autophagic vacuoles. The histogram indicates the relative level of autophagic vacuoles. (d–i) The expression levels of autophagy-related proteins in two TSCC cells were measured by Western blotting. The histograms indicate the quantification of relative level of proteins. The quantitative data are shown as the mean ± SD of 3 independent experiments. ^∗^*p* < 0.05, ^∗∗^*p* < 0.01, and ^∗∗∗^*p* < 0.001.

Autophagy is an intracellular process in which cytoplasmic components are transported to the lysosome for degradation. It is characterized by the increase in acidic vesicular organelles (AVOs) [23]. Our previous study has demonstrated that PLB can induce autophagy in TSCC cells *in vitro*. To investigate whether autophagy participates in the synergistic effect, we examined the autophagy levels in TSCC cell lines. Firstly, the membrane acidotropic dye probe (LysoTracker Red DNA-99) was used to label cellular acidic compartments, such as autolysosomes and lysosomes. As shown in Figure 3(a), compared with cells exposed to cisplatin or PLB alone, cells exposed to cisplatin combined with PLB significantly increased AVO accumulation in the cytoplasmic perinuclear region. Secondly, transmission electron microscopy (TEM) was used to examine the formation of autophagosome. We observed a high level of lysosome and autophagosome formation following the combination treatment in CAL27 cells (Figures 3(b) and 3(c)). In contrast, in the control group, lysosomes and autophagosomes were difficult to observe. Lastly, we also tested the expression levels of autophagy-related proteins by Western blotting. Beclin-1 plays an essential role in the initiation of autophagy. The results showed that the production of Beclin-1 was significantly upregulated in the combination treatment group compared with the CDDP or PLB group alone (Figures 3(d) and 3(e)). When autophagy is inhibited, p62 will accumulate, and when there is autophagy flux, p62 will decrease [24]. Compared with CDDP or PLB treatment alone, the combination treatment dramatically downregulated the expression level of p62 (Figures 3(d) and 3(f)). The transformation of LC3-I to LC3-II is considered to be a marker of autophagy in mammals [25]. We found that the conversion of LC3-I to LC3-II was obviously increased in the combination treatment group compared with the CDDP or PLB treatment alone (Figures 3(d) and 3(g)). In addition, the AKT/mTOR signaling pathway plays a crucial role in mediating cellular autophagy, whereas the activation of mTOR inhibits autophagy [26]. As shown in Figures 3(d), 3(h), and 3(i), compared with the CDDP or PLB treatment alone, the combination treatment resulted in a significant reduction in p-AKT and further resulted in a decrease in p-mTOR.

**Figure 4:**
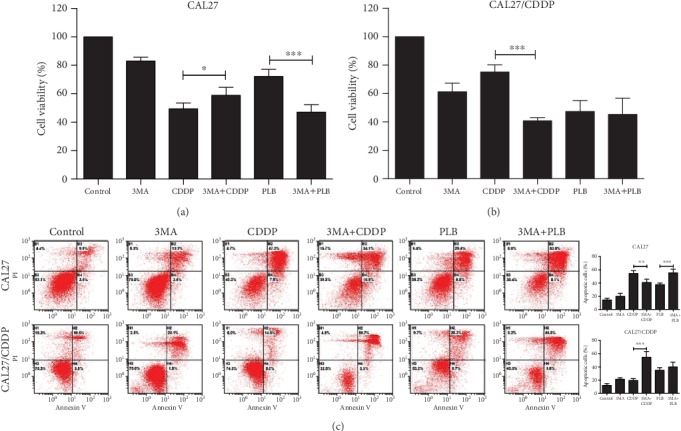
The role of autophagy induced by CDDP and PLB. CAL27 and CAL27/CDDP cells were exposed to autophagy inhibitor 3-MA (2 mM), PLB (5 *μ*M) and CDDP (16.7 *μ*M) alone, and pretreatment with 3-MA for 1 h prior to PLB or CDDP. (a, b) Cell viability was measured by CCK-8 assay. (c) Flow cytometry analysis of Annexin V and PI staining of apoptotic cells. The histograms indicate the quantification of the early and late apoptotic cells. ^∗^*p* < 0.05, ^∗∗^*p* < 0.01, and ^∗∗∗^*p* < 0.001.

Autophagy has been claimed to play a paradoxical role in controlling cell death and survival in response to various stimuli [27]. Previous studies have reported that induction of autophagy could promote cisplatin-induced chemoresistance in osteosarcoma [28]. To investigate the role of CDDP- or PLB-induced autophagy, the inhibitor 3-methyladenine (3-MA, inhibitor of early autophagy/LC3-II accumulation) was used to pretreat TSCC cells. As shown in Figure 4(a), our results indicated that pretreatment with 3-MA reduced the inhibitory effect of CDDP on CAL27 cell viability. In contrast, 3-MA enhanced the inhibition of CDDP on CAL27/CDDP cell viability (Figure 4(b)). This finding suggests that autophagy in CAL27/CDDP cells likely serves as a marker for cisplatin resistance. In addition, we found that 3-MA enhanced the inhibitory effect of PLB on TSCC cell viability (Figures 4(a) and 4(b)). As expected, the results of flow cytometry for apoptosis also reflected a similar phenomenon (Figure 4(c)). All of the above findings indicate that PLB increases CDDP-induced autophagy in TSCC cells, whereas PLB-induced autophagy promotes survival of CAL27 and CAL27/CDDP cells. Moreover, autophagy in drug-resistant cells may be one of the causes of cell resistance.

### 3.4. PLB Inhibits the Viability and Growth of TSCC Cells by Increasing the Production of Intracellular ROS

**Figure 5:**
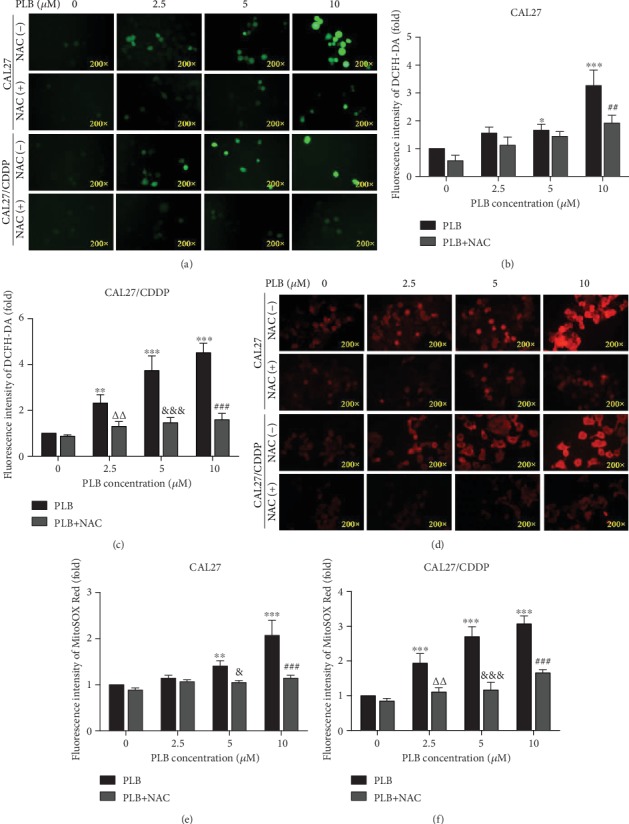
PLB induces the production of intracellular ROS. CAL27 and CAL27/CDDP cells were treated with different doses of PLB in the presence or absence of 5 mM NAC. (a–c) Cells were stained with 10 *μ*M DCFH-DA, and the level of ROS was determined by fluorescence microscopy. The mean fluorescence intensity of ROS was shown in histograms. (d–f) Cells were stained with 4 *μ*M MitoSOX Red dye, and the level of ROS was measured by fluorescence microscopy. The mean fluorescence intensity of ROS was shown in histograms. ^∗^*p* < 0.05, ^∗∗^*p* < 0.01, and ^∗∗∗^*p* < 0.001 vs. control; *^ΔΔ^p* < 0.01 vs. PLB (2.5 *μ*M); ^&^*p* < 0.05 and ^&&&^*p* < 0.001 vs. PLB (5 *μ*M); ^##^*p* < 0.01 and ^###^*p* < 0.001 vs. PLB (10 *μ*M).

ROS production is associated with PLB-mediated anticancer effects [9]. To verify the effect of PLB on ROS in TSCC cells, the ROS scavenger NAC was used in our experiment. Firstly, we used the fluorescent dye DCFH-DA to assess cellular ROS levels. As shown in Figures 5(a)–5(c), PLB treatment alone caused a concentration-dependent increase in DCFH-DA fluorescence signal in CAL27 and CAL27/CDDP cells. However, such elevation of ROS was sufficiently blocked by 1 h pretreatment with ROS scavenger NAC. In addition, we also used the oxidation-sensitive red fluorescence dye MitoSOX Red, a mitochondrial targeting probe sensitive to mitochondrial ROS. As expected, PLB also caused an increase in MitoSOX Red fluorescence in a concentration-dependent manner, which was also scavenged by NAC (Figures 5(d)–5(f)). These results reveal that PLB induces intracellular ROS generation in CAL27 and CAL27/CDDP cells.

**Figure 6:**
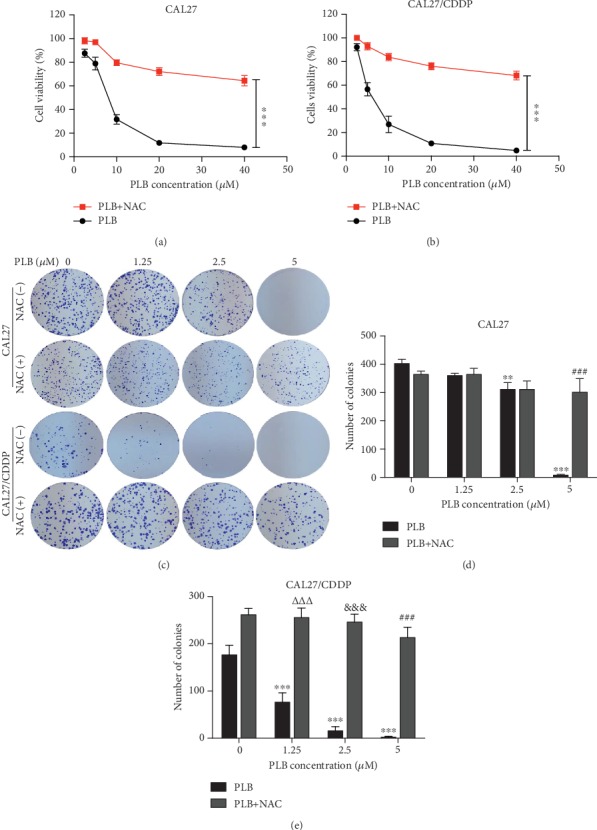
PLB inhibits the viability and growth of TSCC cells by increasing the production of intracellular ROS. CAL27 and CAL27/CDDP cells were treated with different doses of PLB in the presence or absence of 5 mM NAC. (a, b) Cell viability was measured by CCK-8 assay. ^∗∗∗^*p* < 0.001. (c–e) Representative images and quantification of colony formation assay. ^∗∗^*p* < 0.01 and ^∗∗∗^*p* < 0.001 vs. control; *^ΔΔΔ^p* < 0.001 vs. PLB (1.25 *μ*M); ^&&&^*p* < 0.001 vs. PLB (2.5 *μ*M); ^###^*p* < 0.001 vs. PLB (5 *μ*M).

Next, we further determined whether pretreatment with ROS scavenger NAC can protect TSCC cells from the cytotoxic effects of PLB. We evaluated the cytotoxicity of PLB alone or in combination with NAC on TSCC cells by CCK-8 assay. As shown in Figures 6(a) and 6(b), compared with the PLB treatment alone, the viability of the two TSCC cell lines was significantly elevated in the group pretreated with NAC. Furthermore, we also observed the growth of TSCC cells via clone formation analysis. Interestingly, cells incubated with NAC 1 h prior to PLB treatment substantially increased the number of clones (Figures 6(c)–6(e)). These observations indicate that PLB can inhibit the viability and growth of CAL27 and CAL27/CDDP cells via regulating intracellular ROS.

### 3.5. Plumbagin Enhances the Cytotoxicity of Cisplatin to TSCC Cells by Regulating ROS-Mediated JNK and AKT/mTOR Signaling Pathways

**Figure 7:**
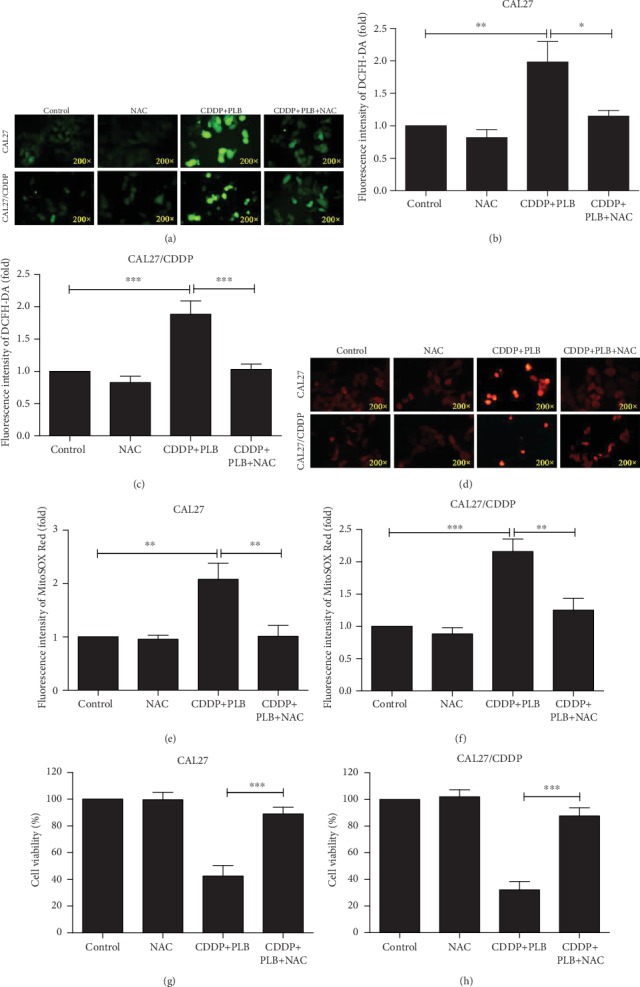
The role of ROS in the synergistic effect. CAL27 and CAL27/CDDP cells were pretreated with NAC (5 mM) for 1 h prior to cotreatment with PLB (5 *μ*M) and CDDP (16.7 *μ*M) for another 24 h. (a–c) Cells stained with 10 *μ*M DCFH-DA, the level of ROS was determined by fluorescence microscopy. The mean fluorescence intensity of ROS was shown in histograms. (d–f) Cells were stained with 4 *μ*M MitoSOX Red dye, and the level of ROS was measured by fluorescence microscopy. The mean fluorescence intensity of ROS was shown in histograms. (g, h) Cell viability was measured by CCK-8 assay. ^∗^*p* < 0.05, ^∗∗^*p* < 0.01, and ^∗∗∗^*p* < 0.001.

**Figure 8:**
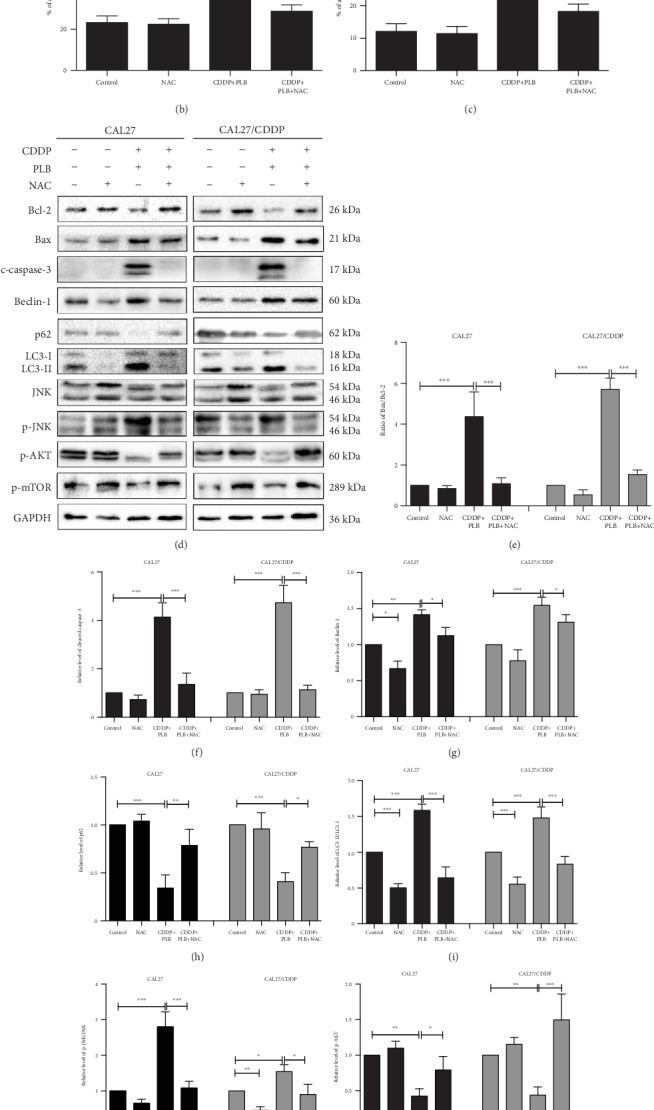
The role of ROS in autophagy, apoptosis, and signaling pathways mediated by the combination treatment. CAL27 and CAL27/CDDP cells were pretreated with NAC (5 mM) for 1 h prior to cotreatment with PLB (5 *μ*M) and CDDP (16.7 *μ*M) for another 24 h. (a–c) Flow cytometry analysis of Annexin V-FITC and PI staining of apoptotic cells. The histograms indicate the quantification of the early and late apoptotic cells. (d–l) The expression levels of proteins involved in autophagy, apoptosis, JNK signaling pathway, and AKT/mTOR signaling pathway were measured by Western blotting. The histograms indicate the relative expression levels of proteins. The quantitative data are shown as the mean ± SD of 3 independent experiments. ^∗^*p* < 0.05, ^∗∗^*p* < 0.01, and ^∗∗∗^*p* < 0.001.

Subsequently, we investigated whether ROS accumulation is a necessary event in the synergistic effect. As shown in Figures 7(a)–7(c), CAL27 and CAL27/CDDP cells were treated with a combination of CDDP and PLB, resulting in a significant increase in ROS production compared to the control. However, the increase in ROS production in TSCC cells was effectively inhibited after pretreatment with ROS scavenger NAC. At the same time, a similar phenomenon was observed in the MitoSOX Red fluorescence assay (Figures 7(d)–7(f)). Cotreatment with NAC rescued the combination treatment-induced cytotoxicity in both CAL27 and CAL27/CDDP cells (Figures 7(g) and 7(h)). Flow cytometry also showed that pretreatment with NAC significantly reversed apoptosis induced by the combination treatment in both TSCC cells (Figures 8(a)–8(c)). Similarly, these findings elucidated the vital role of ROS in the synergistic effect of PLB and CDDP.

Accumulating evidence suggests that excessive amounts of ROS may change cell signaling pathways for autophagy and apoptosis [29, 30]. We revealed that the expression of Bax and cleaved caspase-3 induced by PLB combined with CDDP can be induced in response to ROS, and increased ratio of LC3-II/LC3-I as well as Beclin-1 can be inhibited by the cotreatment with NAC (Figures 8(d), 8(g), and 8(i)). Next, we tested the connection between ROS accumulation and JNK signaling pathway. We found that pretreatment with NAC markedly reversed JNK phosphorylation induced by the combined treatment in CAL27 and CAL27/CDDP cells (Figures 8(d) and 8(j)). Furthermore, many evidences suggest that ROS plays an important role in mediating the AKT/mTOR signaling pathway [31, 32]. Therefore, we explored whether the combination treatment can downregulate the AKT/mTOR signaling pathway via regulating the ROS in TSCC cells. Our results indicated that NAC partially blocked the phosphorylation of AKT and mTOR activated by the combination treatment (Figures 8(d), 8(k), and 8(l)). Based on these findings, the apoptosis and autophagy induced by the combination of PLB and CDDP in TSCC cells can be mediated by the accumulation of intracellular ROS through the JNK and AKT/mTOR signaling pathways.

### 3.6. Plumbagin and Cisplatin Combination Inhibited TSCC Xenograft Tumor Growth In Vivo

**Figure 9:**
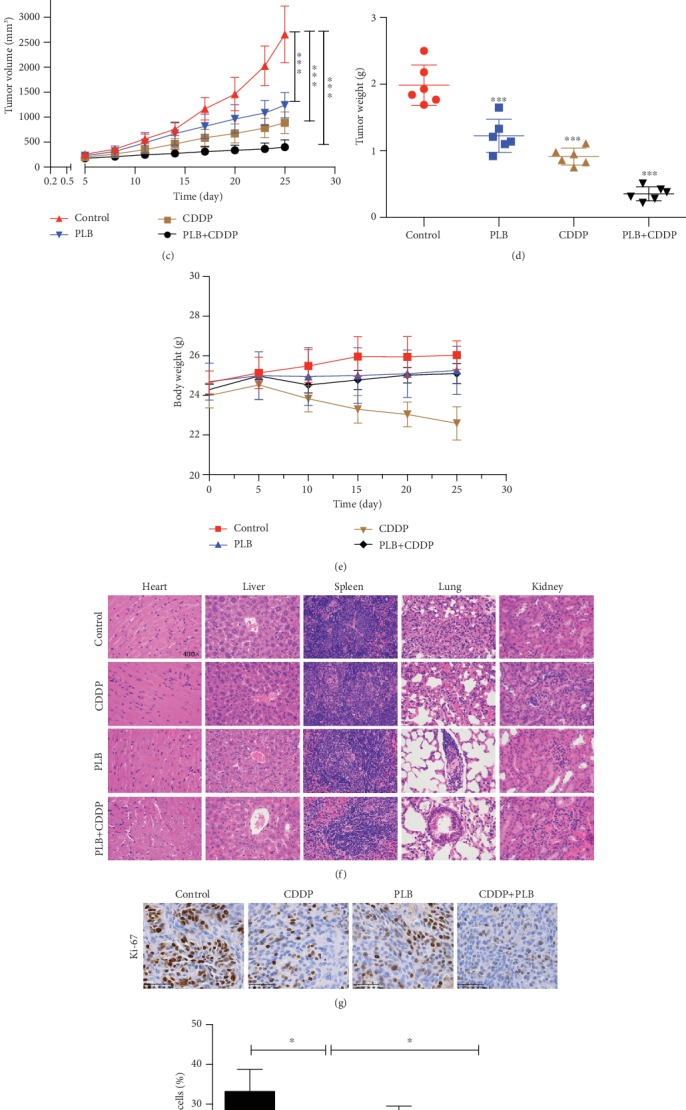
Antitumor efficacy of PLB in combination with CDDP in CAL27/CDDP xenograft tumor models *in vivo*. (a) CAL27/CDDP cells were subcutaneously injected to the right flanks of BALB/C nude mice. The mice were treated with PLB, CDDP, or their combination as described in Methods. (b) The mice were sacrificed after the last treatment, and tumor xenografts were carefully dissected and presented. (c) Volumes of the xenograft tumors after administration of PLB, CDDP, or their combination. ^∗∗∗^*p* < 0.001. (d) Tumor weights were measured at the end of the experiment. ^∗∗∗^*p* < 0.001 vs. Control. (e) Body weights were recorded after administration of PLB, CDDP, or their combination. (f) Normal organs were sectioned and stained with H&E after treatment with PLB, CDDP, or their combination. (g) Representative immunohistochemical staining of Ki-67 from tumor sections via IHC. (h) Statistical analyses of the expression of Ki-67 in different groups. ^∗^*p* < 0.05.

Based on the synergistic inhibition of PLB and CDDP combination treatment on TSCC cells *in vitro*, we estimated whether similar therapeutic effects could occur in a subcutaneous cisplatin-resistant xenograft model. CAL27/CDDP cells were subcutaneously injected into the immunodeficient mice. After 21 days of treatment, we found that all treatment groups showed an effective inhibition on the growth of tumor (Figures 9(a)–9(d)). However, PLB combined with CDDP treatment exhibited the greatest inhibitory effects on tumor volume and weight (Figures 9(a)–9(d)). Interestingly, CDDP treatment alone caused a significant decrease in body weight, whereas the combination treatment reversed the phenomenon (Figure 9(e)). This body weight curves indicated that PLB combined with CDDP was less system toxic than CDDP treatment alone. Moreover, hematoxylin and eosin (H&E) staining further proved that the combination treatment group did not cause major organ-related toxicities (Figure 9(f)). Furthermore, IHC staining showed that PLB combined with CDDP significantly repressed expression of Ki-67 (Figures 9(g) and 9(h)). Taken together, these results suggest that PLB combined with CDDP is a promising strategy for overcoming cisplatin resistance of TSCC while reducing toxicities comparing to cisplatin treatment alone.

## 4. Discussion

Cisplatin is used in the first-line therapy of advanced TSCC, but resistance and toxicity limit its clinical benefits. Recently, in order to enhance the chemosensitivity of tumors to cisplatin, phytochemicals have received more and more attention due to their biological activity and low toxic effects. Anticancer activities associated with PLB have been reported in various cancer types. We have previously shown that PLB has profound antiproliferative effects on TSCC cells *in vitro* and *in vivo* [11, 12]. Therefore, we were interested in whether PLB could enhance the anticancer effect of cisplatin *in vitro* and in established TSCC xenograft tumor models. Herein, we show that PLB displays growth inhibitory effect in both CAL27 cells and cisplatin-resistant CAL27/CDDP cells. In addition, we also found that the combination of low concentrations of PLB and CDDP exhibited synergistic inhibitory effects (CI < 1) on both CAL27 and cisplatin-resistant CAL27/CDDP cells. Interestingly, the synergistic inhibition of PLB and CDDP was more pronounced in CAL27/CDDP cells. Other authors have investigated the role of plumbagin in enhancing thalidomide and bortezomib in multiple myeloma cells [20]. However, the treatment of PLB combined with CDDP has not been reported.

Although several mechanisms have been elucidated to enhance the anticancer effect of cisplatin, it is still not completely understood. Apoptosis is a mechanism of programmed cell death regulated by cellular signaling pathways [33]. Cancer cell escape from apoptosis is one of the resistance mechanisms of chemotherapy [34]. It frequently exhibits expression dysregulation of the Bcl-2 family proteins, including overexpression of antiapoptotic proteins and decreased expression of proapoptotic proteins, which are closely related to apoptosis resistance and chemotherapy resistance [35]. Thus, targeting the apoptotic pathway and further increasing the sensitivity of cancer cells to cisplatin-induced apoptosis are considered to be an effective adjuvant for CDDP-based chemotherapy. In the present study, our results clearly show that PLB and CDDP cotreatment induces a series of cellular events, including increased nuclear fragmentation, increased percentage of apoptotic cells, and characteristic apoptotic protein expression (caspase-3 activation). At the same time, combination treatment also downregulated Bcl-2 and upregulated Bax expression, which is closely related to mitochondrial apoptosis resistance and chemoresistance.

Apart from apoptosis, autophagy plays a paradoxical role in determining the fate of cancer cells. It has been reported that autophagy protects cells from oxidative stress and promotes cancer cell resistance to chemotherapy [36]. Paradoxically, autophagy is also recognized as a useful strategy for inhibiting tumor growth and inducing cell death in chemoresistance [37]. Studies have shown that autophagy can promote cell death as a cytotoxic mechanism when cisplatin does not trigger apoptotic responses in apoptotic-resistant cells [38]. In the present study, the combination of PLB and CDDP treatment induced autophagy, as evidenced by the accumulation of AVOs and autophagosomes as well as the upregulation of LC3-II, etc. The AKT/mTOR signaling pathway plays a key regulatory role in cell functions such as cell growth, apoptosis, and autophagy [39]. Our results showed that the PLB and CDDP cotreatment significantly inhibited the expression of p-AKT and p-mTOR, compared with either PLB and CDDP treatment alone. However, the inhibition of autophagy by the autophagy inhibitor 3-MA could not reverse cell death induced by PLB treatment alone. Notably, we found that, unlike CAL27 cells, CDDP caused protective autophagy in CAL27/CDDP cells. These findings indicate that the increased resistance of CAL27/CDDP cells to cisplatin is closely related to the activation of self-defensive autophagy. In support of our data, Chu et al. demonstrated that autophagy inhibitor bafilomycin A1 could increase the sensitivity of TSCC cells to cisplatin [40].

ROS is an important signaling molecule in various vital cellular processes, including cell apoptosis, autophagy, proliferation, and differentiation [41]. Many studies have found that excessive accumulation of intracellular ROS caused irreversible cellular damage leading to autophagy and/or apoptosis [42, 43]. Chao et al. found that PLB can induce accumulation of ROS in human osteosarcoma, leading to mitochondrial apoptosis [44]. In prostate cancer, PLB induced apoptosis and autophagy through ROS-associated pathway [10]. Herein, we found that PLB caused the generation of ROS in a dose-dependent manner and decreased the cell viability and growth. ROS scavenger NAC remarkably inhibited the ROS generation induced by PLB and reversed the cell viability and growth inhibited by PLB. These results are consistent with other findings demonstrating that ROS is closely related to PLB-induced cytotoxicity.

ROS is a key mediator of CDDP-induced cytotoxicity. It has been reported that oxidative stress is involved in CDDP-induced apoptosis, and antioxidants can eliminate its cytotoxic effects [45]. He et al. showed that Curcuminoid WZ35 exerted a synergistic effect with cisplatin on inducing ROS production in gastric cancer [46]. Therefore, we explored whether ROS participate in the synergistic effect of PLB and CDDP. We found that the combination of PLB and CDDP resulted in a significant increase in intracellular ROS. However, pretreatment with NAC can completely reverse the accumulation of ROS, autophagy, and apoptosis caused by combination treatment, while retaining the viability of TSCC cells. In addition, we also evaluated changes in apoptosis- and autophagy-related proteins with or without pretreatment with ROS scavenger NAC. We found that NAC downregulated the ratio of Bax/Bcl-2 induced by the combined treatment and resulted in a decrease in caspase-3 expression. NAC also decreased the expression of Beclin-1 and the conversion of LC3-I to LC3-II induced by the combined treatment. Our data showed that cotreatment with PLB and CDDP induces ROS generation in both CAL27 and CAL27/CDDP cells and that scavenged ROS by NAC almost completely suppresses cell death. The evidence we provided supports a close relationship between increased levels of ROS and the synergistic effect of PLB and CDDP.

Studies have suggested that activation of ROS/JNK and inhibition of the AKT/mTOR signaling pathway can effectively induce apoptosis and autophagy in cancer cells [31]. Therefore, we have further explored the underlying mechanism of combination treatment to induce apoptosis and autophagy. Our present results showed that the combined treatment induced the accumulation of ROS that promoted the activation of JNK and the inhibition of AKT/mTOR in both CAL27 and CAL27/CDDP cells. In sum, these findings reveal that PLB combined with CDDP is an effective combination for inhibiting TSCC growth by inducing ROS accumulation affecting the JNK and AKT/mTOR pathway.

Finally, we further examined the combination effects of PLB and CDDP *in vivo* by constructing the nude mice xenograft models. In our study, we found that the combination treatment group exhibited the greatest antitumor effects *in vivo*. Furthermore, it is worth noting that PLB reversed the body weight loss caused by cisplatin treatment. And H&E staining of organs suggested that the combined treatment group did not induce major organ-related toxicities. So we can conclude that this combination might be a relatively effective and safe regimen for TSCC.

## 5. Conclusions

In summary, low levels of ROS cause chemoresistance in TSCC cells. Thus, increasing intracellular ROS levels may represent a novel target to improve chemosensitivity. In this work, our findings highlight that PLB, as a novel potential chemotherapy sensitizer, can overcome chemotherapy resistance in patients with TSCC by generating ROS.

## Data Availability

The data used to support the results in this study are included in the article. The materials for the current study are available from the corresponding author upon reasonable request.

## Abbreviations

TSCC: Tongue squamous cell carcinoma
PLB: Plumbagin
CDDP: Cisplatin
ROS: Reactive oxygen species
FBS: Fetal bovine serum
DMEM: Dulbecco's modified Eagle's medium
NAC: N-Acetylcysteine
3-MA: 3-Methyladenine
DAPI: 4′6′-Diamidino-2-phenylindole
PI: Propidium iodide
RIPA: Radioimmunoprecipitation assay
DCFH-DA: 2′,7′-Dichlorofluorescein diacetate
BSA: Bovine serum albumin
CI: Combination index
OD: Optical density.
